# Avenanthramides as digestion-stage modulators: Mechanistic links across the gastrointestinal continuum

**DOI:** 10.1016/j.fochx.2026.104252

**Published:** 2026-07-27

**Authors:** Nima Mohammadi, Amalia G.M. Scannell, Marilú Andrea Silva-Espinoza

**Affiliations:** a UCD Institute of Food and Health, University College Dublin, Belfield, Dublin 4 D04 V1W8, Ireland; b UCD School of Agriculture and Food Science, University College Dublin, Belfield, Dublin 4 D04 V1W8, Ireland

**Keywords:** Bioaccessibility, Dihydroavenanthramides, Metabotype variability, Resistant starch modulation, Gut microbiota

## Abstract

Oat avenanthramides (AVNs) are phenolic alkaloids unique to oats and are widely recognised for their antioxidant and anti-inflammatory properties. However, human studies consistently report very low oral bioavailability (<2%), highlighting a disconnect between dietary intake and systemic exposure. This review presents a digestion-stage-resolved conceptual framework integrating current evidence on AVN fate during gastrointestinal digestion, including matrix entrapment, digestive release, limited absorption, microbial biotransformation, and interindividual metabotype variability. AVN bioaccessibility is influenced by food matrix composition, processing conditions, and enzymatic activity. Evidence indicates that AVNs may modulate starch hydrolysis, glucose transport, and resistant starch formation while undergoing chemical and Phase I transformations. In the colon, microbial metabolism produces dihydroavenanthramides (DH-AVNs), which may contribute to biological activity in a metabotype-dependent manner. Overall, AVNs appear to exert physiological effects primarily through local gastrointestinal mechanisms, although proposed pathways require confirmation in well-designed human intervention studies.

## Highlights

• Oral bioavailability of oat avenanthramides is extremely low at <2%.
• AVNs show high binding affinity for starch matrices (92.9% to 99.8%).
• AVNs inhibit SGLT1 by ≥75% and block GLUT2 glucose uptake at 150 μM.
• AVN-starch interactions increase resistant starch content by up to 4.3-fold.
• *F. prausnitzii* converts AVNs to DH-AVNs in >50% of human metabolizers.

## 1 Introduction

Avenanthramides (AVNs) are low-molecular-weight phenolic alkaloids found almost exclusively in oats (Zhang, Ni, et al., 2020). While they function as plant defense compounds and bioactive modulators with antioxidant, anti-inflammatory, antipruritic, and cardiometabolic effects in humans, direct clinical evidence remains limited; many of these benefits are proposed to arise from digestion-mediated mechanisms rather than systemic exposure (Ishihara, 2021; Kim, Hwang, et al., 2021; Park et al., 2021). Despite potent *in vitro* effects, translation to *in vivo* outcomes is limited, as human studies consistently reveal extremely low oral bioavailability (<2%) (C. Chen et al., 2022; Shlisky et al., 2025), highlighting a “bioavailability paradox” between intake and systemic exposure. This discrepancy highlights the need to evaluate AVNs through a digestion- and metabolism-focused lens, rather than relying solely on raw-grain concentration or chemical abundance.

Recognising this limitation has shifted research toward digestion-stage–resolved frameworks that consider bioaccessibility, enzymatic and microbial transformations, and post-digestion functionality (C. Chen et al., 2024; Yu et al., 2022; Živković et al., 2025). Within oat matrices, AVNs are often associated with plant cell wall components or embedded in starch–protein networks, meaning that functional potential is determined largely by release from the matrix during digestion (Armeni et al., 2025; C. Chen et al., 2024; Jiménez-Pulido et al., 2022). Processing conditions, matrix architecture, and enzymatic interactions further modulate AVN availability, emphasising the importance of considering food structure in functional assessments. Beyond chemical release, AVNs undergo extensive microbial biotransformation in the colon, producing metabolites such as dihydroavenanthramides (DH-AVNs), which may account for a substantial proportion of post-digestive biological activity (Yu et al., 2022). Interindividual variability in gut microbiota composition makes AVN functionality metabotype-dependent, highlighting the limitation of assuming universal efficacy across populations (Favari et al., 2024; *P.* Wang et al., 2021). AVNs also actively modulate the structural behaviour of the food matrix, influencing starch hydrolysis, glucose release, and resistant starch formation (C. Chen et al., 2024). Collectively, these observations suggest that AVNs may function as digestion-stage-dependent gastrointestinal modulators rather than solely as passive bioactive phytochemicals. However, this interpretation should be regarded as a conceptual model that integrates evidence from food chemistry, digestion studies, cellular models, animal experiments, and the limited number of available human studies, rather than as an established physiological paradigm.

Despite growing research interest, the literature remains fragmented across chemical (Jágr et al., 2020), digestive (Armeni et al., 2025; C. Chen, Li, Chen, Wang and Luo, 2020, Chen, Yao, Wang, Chen and Wang, 2024), microbial (Campbell, 2021; *P.* Wang et al., 2021), and functional domains (Fu et al., 2022; Y. Liu et al., 2024; Zhang, Ni, et al., 2020). Mechanistic understanding of how structurally complex, matrix-embedded AVNs translate into physiological functionality remains limited by these reductionist approaches. Importantly, although previous reviews have comprehensively discussed AVN chemistry, antioxidant activity, bioavailability, or gut microbiota interactions individually, none has systematically integrated matrix entrapment, digestive transformation, intestinal functionality, microbial biotransformation, and interindividual metabotype variability within a single digestion-stage-resolved framework. To highlight the conceptual novelty of the present review, Table 1 compares its scope with previously published AVN reviews and illustrates how the proposed framework unifies these research areas within a single digestion-stage-resolved perspective.

**Table 1:** Comparison of previous reviews on oat avenanthramides and the digestion-stage-resolved framework proposed in the present review.

Review articles	Structure	Bioavailability	Gut microbiota	Digestion-stage framework	Matrix interactions
(Boz, 2015)	Describes basic hydroxycinnamic acid–anthranilic acid conjugates.	Not discussed (only notes clinical efficacy of oral intake but lacks absorption metrics).	Not discussed (briefly mentions colon cancer prevention but omits microbial pathway).	No integrated framework.	Not discussed.
(Perrelli et al., 2018)	Focuses on major AVN * isoforms (A, B and C) and therapeutic potential.	Discusses tissue distribution following absorption.	Not discussed (mentions general systemic “biotransformation rates” in tissue distribution, but lacks any mention of microbes or the gut).	Not discussed.	Not discussed (briefly lists distribution across oat milling fractions and a whole-food clinical pilot, but omits chemical matrix binding/release dynamics).
(W. Wang et al., 2020)	Reviews phenolamide chemistry and classification.	Notes that human absorption and functionality remain poorly understood.	Suggests possible microbial metabolism.	Not discussed.	Not discussed.
(Yu et al., 2022)	Comprehensive overview of AVN nomenclature, chemistry and metabolism (>40 AVNs).	Notes low overall polyphenol bioavailability	Introduces the role of *Faecalibacterium prausnitzii*.	Reviews individual digestive processes but not within a unified framework.	Brief discussion of delivery systems.
(Pretorius & Dubery, 2023)	Detailed review of biosynthesis and chemistry.	Not discussed (only general health benefits of consumption are listed).	Not discussed	No integrated digestion-stage framework.	Minimally noted; mentions they function in concert with dietary fibres for health.
(Xie et al., 2024)	Updates chemical diversity and biological activities.	Discusses plasma concentration peaks and notes human bioavailability is significantly higher than in animal models.	Not discussed	No integrated framework.	Not discussed.
Present review	Integrates structural diversity, E/Z isomerism and matrix-associated structural changes occurring throughout digestion.	Interprets the bioavailability paradox by distinguishing local gastrointestinal exposure from systemic bioavailability within a stage-specific framework.	Integrates microbial biotransformation, DH-AVN formation, and metabotype-dependent variability into AVN functionality.	Provides the first digestion-stage-resolved conceptual framework linking matrix release, gastric stability, intestinal transformation, local functionality, absorption and colonic metabolism.	Systematically integrates evidence on AVN interactions with starch and proteins and their implications for bioaccessibility, glucose regulation and resistant starch formation.

* Note: AVN(s) = Avenanthramide(s); DH-AVN = Dihydro-avenanthramide.

As shown in Table 1, previous reviews have primarily examined individual aspects of AVN chemistry, bioactivity, bioavailability, or gut microbiota, whereas the present review provides a unified mechanistic perspective linking food matrix interactions, gastrointestinal digestion, intestinal functionality, microbial biotransformation, and interindividual variability. Accordingly, the novelty of the present review lies not in proposing new biological mechanisms, but in integrating existing evidence into a digestion-stage-resolved conceptual framework that highlights mechanistic links across the gastrointestinal continuum.

To address these gaps, this review proposes a digestion-stage-resolved conceptual framework that follows the sequential fate of AVNs from food matrix incorporation to colonic microbial metabolism. Rather than introducing a new biological classification of AVNs, this framework synthesises existing mechanistic evidence into a stage-specific model that highlights how matrix architecture, digestive transformation, intestinal interactions, microbial metabolism, and host variability may collectively determine physiological functionality. By organising the available literature within this gastrointestinal continuum, the framework provides a structured perspective for identifying current knowledge gaps, evaluating the strength of existing evidence, and guiding future mechanistic and clinical investigations.

## 2 AVN structure and matrix association

At ingestion, AVNs enter the digestive tract as phenolic alkaloids defined as conjugates of an anthranilic acid moiety and a phenylalkenoic acid moiety linked through a covalent amide (pseudo-peptide) bond (Woolman & Liu, 2022; Zhang, Zhao, et al., 2020). AVNs comprise a structurally diverse group, with over 40 isoforms identified in oats to date (Trabalzini et al., 2022; Xie et al., 2024). The predominant isoforms, AVN-A (2p), AVN-B (2f), and AVN-C (2c), differ in hydroxyl and methoxy substitution patterns on the phenylalkenoic acid ring. AVN-A contains a single para-hydroxyl group derived from *p*-coumaric acid, AVN-B contains a 4′-hydroxyl and 3′-methoxy substitution corresponding to a ferulic acid (guaiacyl) structure, and AVN-C contains ortho-dihydroxyl groups at the 3′ and 4′ positions corresponding to a caffeic acid (catechol) structure (Kim, Hwang, et al., 2021; Yu et al., 2022).

Based on side-chain characteristics, AVNs are further classified into C-type forms containing cinnamic acid derivatives with one double bond and A-type forms containing avenalumic acid derivatives with two double bonds (W. Wang et al., 2020). Within the food matrix, AVNs are present as aglycones or as glycosylated derivatives, including hexosides and glucosides, and exhibit geometric isomerism, predominantly in the naturally occurring E (trans) configuration, although Z (cis) isomers are also detected (Jágr, Dvořáček, Čepková and Doležalová, 2020, Jágr et al., 2024; Sumayya et al., 2022).

**Fig. 1:**
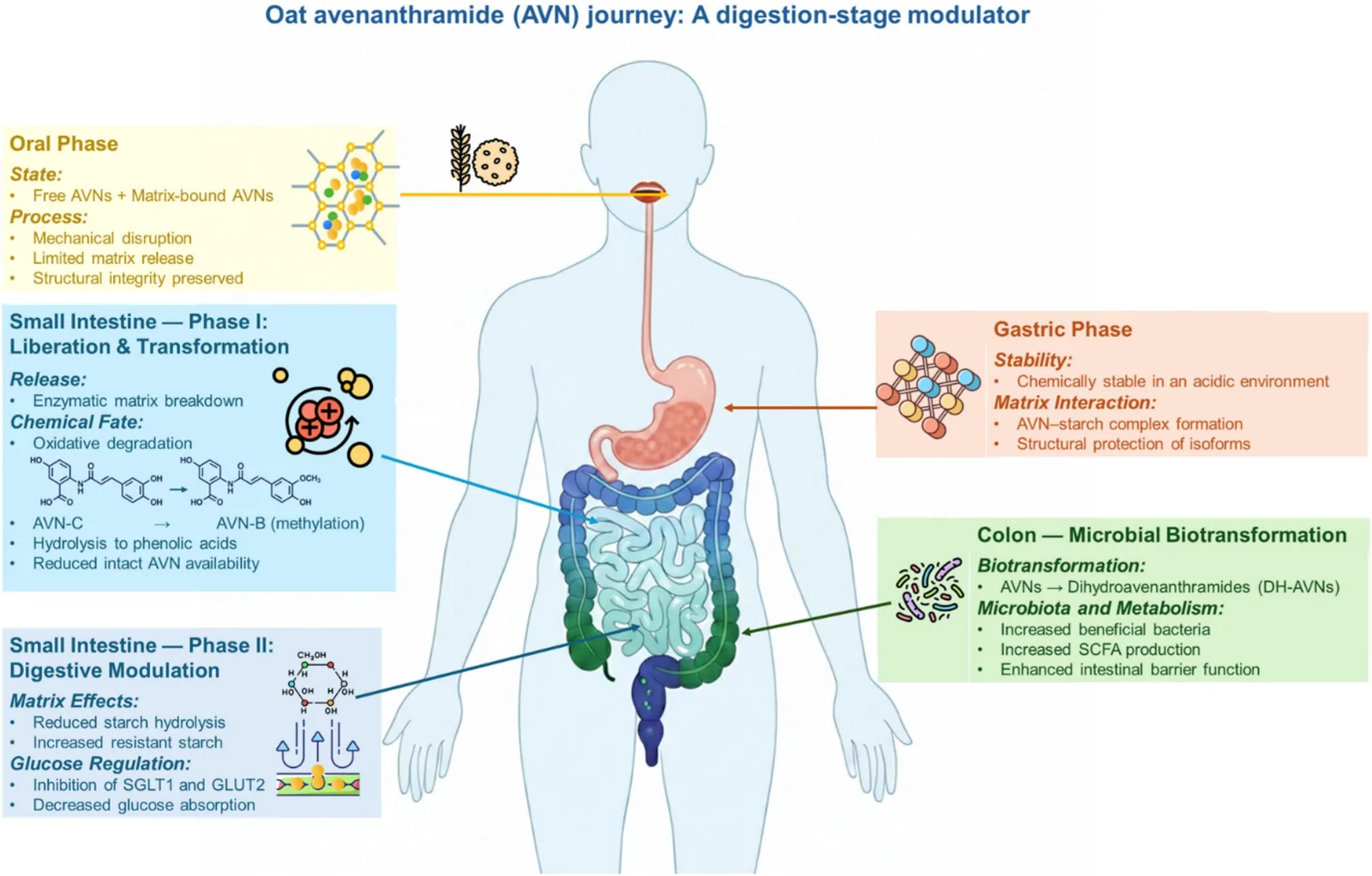
Schematic representation of the metabolic fate of oat avenanthramides (AVNs) during human digestion. The figure traces the chemical stability, enzymatic transformation, and microbial biotransformation of AVNs across the oral, gastric, intestinal, and colonic environments**.**

Physically, AVNs exist in both free (soluble) and insoluble-bound states (Aiello et al., 2021; Hakkola et al., 2021) (Table 2 and Fig. 1). A substantial proportion is associated with macromolecules in the plant cell wall, leading to matrix entrapment within structural regions of the oat grain, including the aleurone, germ, and endosperm (Brzozowski et al., 2022; Feng et al., 2022; Hafez-Ghoran et al., 2025). In the bound state, AVNs are covalently linked to cellulose, hemicellulose, or lignin *via* ester and ether linkages. Their retention within the oat matrix is further reinforced by hydrogen bonding, hydrophobic interactions, and electrostatic forces within starch–protein networks, collectively limiting bioaccessibility at the point of consumption (Călinoiu & Vodnar, 2020; Shlisky et al., 2025). These observations suggest that matrix entrapment may influence AVN bioaccessibility by regulating the extent and timing of their release during digestion. However, the molecular nature of these interactions remains incompletely understood, and current evidence is derived predominantly from *in vitro* digestion models rather than *in vivo* studies. Consequently, the physiological relevance of matrix entrapment requires further experimental validation.

**Table 2:** Comparative digestive fate, bioaccessibility, and functional effects of avenanthramides (AVNs) across the human gastrointestinal tract.

Digestive stage	AVN form & structure	Chemical/Physical fate	Interactions & bioaccessibility	Functional/Physiological effects	Isoform-specific notes	References
Entry into the digestive tract	Aglycones or glycosides (hexosides, glucosides), C-type (cinnamic acid derivatives), A-type (avenalumic acid derivatives), E/Z isomers	Exists in free (soluble) or bound (cell wall-linked) forms; bound *via* ester/ether linkages; hydrogen bonding, hydrophobic/electrostatic interactions	Matrix entrapment in aleurone, germ, endosperm; limited bioaccessibility at ingestion	None immediate; structural entrapment limits early release	AVN-A (2p), AVN-B (2f), AVN-C (2c); differ by hydroxyl/methoxy substitutions on phenylalkenoic ring	(Brzozowski et al., 2022; Feng et al., 2022; Hafez-Ghoran et al., 2025; Kim, Hwang, et al., 2021; Trabalzini et al., 2022; W. Wang et al., 2020; Woolman & Liu, 2022; Xie et al., 2024; Yu et al., 2022; T. Zhang, Zhao, et al., 2020)
Oral & gastric digestion (pH 7.0 → 2–3)	Native AVNs	Chemically stable; amide bond intact; minimal degradation under acidic to near-neutral conditions	Redistributes in the food matrix; binds starch and proteins (binding rates 92.9–99.8%); “structural shield” formation	Protects sensitive isoforms (*e.g.*, AVN-C) from early degradation; preserves delivery to the small intestine	AVN-C shows only ∼6% loss at pH 6.0 over 50 min; high binding affinity to starch protects labile isoforms	(Armeni et al., 2025; Chen et al., 2020; Chen et al., 2022; Chen et al., 2024; Yu et al., 2025)
Small intestinal phase I (liberation & chemical fate, pH 7–7.6)	Free and matrix-bound AVNs	Weakly alkaline environment promotes oxidative degradation; enzymatic hydrolysis is possible; Phase I metabolism (oxidation, reduction, hydrolysis, methylation)	Partial release from starch/protein complexes; enzymatic liberation enhanced by endoxylanases, cellulase, xylanase	Hydroxycinnamic and anthranilic acids may form; AVN-C converted to AVN-B *via* COMT; minor derivatives (*e.g.*, AVN-D) can increase	AVN-C is most sensitive to oxidative degradation; AVN-A/B is more stable; enzymatic methylation reduces catechol reactivity	(Chen et al., 2020, Chen et al., 2022, Jiménez-Pulido et al., 2022, Nagaoka et al., 2024, Pretorius & Dubery, 2023, Yu et al., 2025)
Small intestinal phase II (functional modulation)	Released AVNs	Interactions with digestive enzymes; limited absorption	Residual AVN-starch complexes reduce α-amylase access, increasing resistant starch	Inhibit SGLT1 and GLUT2 transporters → lower glucose uptake; dose-dependent IC50 (AVN-B 161.8 μM, AVN-C 227.8 μM)	AVNs may contribute to local modulation of starch digestion and glucose uptake under specific experimental conditions; effective at physiological intestinal concentrations (∼150 μM from oatmeal)	(Aleixandre & Rosell, 2022; Chen et al., 2024; Mathews & Chu, 2025; Zhouyao et al., 2022)
Absorption	Native AVNs	Low paracellular diffusion; limited passive transport; P-gp and MRP2 efflux; Phase II metabolism (sulphate/glucuronide conjugates)	Restricted epithelial permeability; low solubility; minor fraction enters systemic circulation (<2%)	Indirect systemic effects *via* Nrf2 activation; upregulation of antioxidant enzymes (SOD, catalase, glutathione)	High molecular weight (∼300–330 g/mol) and amide bond limit transport; conjugates may act as reversible reservoirs	(Armeni et al., 2025; Baik et al., 2025; Chen et al., 2020; Hakkola et al., 2021; Maliar et al., 2023; Paudel et al., 2021; Roumani et al., 2020; Zarrelli & Longobardo, 2025; Zhang, Ni, et al., 2020)
Colonic phase (microbial biotransformation)	Undigested AVNs (∼95%)	Microbial reduction of C7′–C8′ double bond → DH-AVNs; inter-individual variability due to microbiome composition	*Faecalibacterium prausnitzii* mediates conversion; prebiotics/exercise may enhance metabolizer status	DH-AVNs exhibit stronger bioactivity (anticancer, anti-inflammatory); modulate gut microbiota (↑*Akkermansia*, ↑Firmicutes, ↓*Escherichia–Shigella*); increase SCFA production; reinforce barrier integrity; regulate bile acid/lipid metabolism	DH-2c strongest pro-apoptotic effect; peak plasma concentrations 10–15 h post-ingestion; metabolizers (∼50%), non-metabolizers (∼20%)	(Campbell, 2021; Fu et al., 2022; Liu et al., 2023; Y. Liu et al., 2024; *P.*Wang et al., 2021; Z. Wang et al., 2024; Ye et al., 2025)

## 3 Oral and gastric digestion

During the oral (pH ∼7.0) and gastric (pH 2.0–3.0) phases of digestion, AVNs remain chemically stable, with their molecular structure largely preserved under acidic to near-neutral conditions (C. Chen et al., 2020; Yu et al., 2025). The covalent amide bond linking the anthranilic acid and phenylalkenoic acid moieties remains intact, preventing structural transformation during these early stages. This stability is largely attributed to the low dissociation constant (pKa) of the carboxyl group, which maintains the molecule predominantly in a soluble, monoanionic form, and to the un-ionised state of the phenolic hydroxyl groups, which reduces susceptibility to radical-driven oxidation (C. Chen et al., 2022; Sumayya & Muraleedharan, 2024). AVN-C (2c), in particular, suggests minimal degradation at slightly acidic conditions (pH 6.0), with losses of only ∼6% over 50 min (C. Chen et al., 2022), highlighting the relative resilience of this isoform under near-neutral conditions.

Concurrently, AVNs begin to redistribute within the food matrix, transitioning from their native entrapment in plant cell walls to form complex associations with macronutrients such as starch and proteins (Armeni et al., 2025). As starch granules hydrate and gelatinise, AVNs exhibit a remarkably high binding affinity for the starch matrix, with measured binding rates ranging from 92.9% to 99.8% (C. Chen et al., 2024). This association is facilitated by their structural characteristics, including high molecular weight, peptide bonds, and additional amino groups, which make AVNs more prone to encapsulation within starch granules than simpler phenolic acids. The resulting AVN-starch complexes provide both physical and chemical protection, isolating sensitive isoforms such as AVN-C (2c) from the surrounding environment (C. Chen et al., 2024). These results suggest that AVN–starch interactions may contribute to protecting susceptible AVN isoforms during the early stages of digestion and may influence the timing of their release. Nevertheless, this protective effect has been demonstrated primarily under simulated digestion conditions, and its physiological significance in humans remains uncertain.

## 4 Small intestinal phase I – liberation & chemical fate

The fate of AVNs in the small intestine is influenced by their interactions with the food matrix, the local pH, and host-derived metabolic enzymes. AVNs released from the gastric matrix may still be partially associated with starch or protein complexes, which can influence their subsequent enzymatic hydrolysis and absorption. Release of bound AVNs into the intestinal lumen requires enzymatic or biotechnological techniques leading to the disruption of the matrix prior ingestion (Yu et al., 2025). For example, germination activates endogenous endoxylanases that degrade cell wall arabinoxylans, significantly increasing AVN bioaccessibility (Jiménez-Pulido et al., 2022). Likewise, food-grade enzymes such as cellulase and xylanase can enhance the release of bound phenolic alkaloids by up to fivefold. These observations indicate that AVN bioaccessibility is influenced by food processing and enzymatic disruption of the food matrix. However, most evidence originates from controlled processing experiments, and the relative contribution of endogenous digestive enzymes *in vivo* remains insufficiently characterised.

Once liberated, AVNs are chemically sensitive to the weakly alkaline environment of the small intestine (pH 7.0–7.6), promoting oxidative degradation and reducing intact compound availability (C. Chen et al., 2020, Chen et al., 2022). Stability varies among isoforms, with AVN-C (2c) being highly labile because its ortho-dihydroxy moiety can form a dianion, increasing susceptibility to oxidative degradation (Shlisky et al., 2025). By contrast, AVN-A (2p) and AVN-B (2f) are more stable, exhibiting higher bioaccessibility. This highlights that minor structural differences between isoforms can profoundly influence intestinal availability, a factor often overlooked in *in vitro* studies that treat AVNs as a uniform group. Residual starch interactions may further restrict enzymatic release, reinforcing the importance of considering matrix effects in digestive modelling (C. Chen et al., 2024).

In parallel with chemical degradation, AVNs undergo Phase I enzymatic modifications that influence their chemical structure, polarity, and subsequent absorption (ul Amin Mohsin et al., 2024). Current evidence indicates that intestinal Phase I metabolism of AVNs remains incompletely characterised, and the enzymes responsible for their biotransformation have not been conclusively identified. During intestinal transport, AVNs are hydrolysed to hydroxycinnamic acids (caffeic, ferulic, or *p*-coumaric acid) and anthranilic acid derivatives (Nagaoka et al., 2024), but the hydrolases responsible for this cleavage remain largely unknown. Although intestinal hydrolases such as carboxylesterase 2 (CES2) and arylacetamide deacetylase (AADAC) possess broad substrate specificity toward ester- and amide-containing xenobiotics, direct evidence that these enzymes catalyse AVN hydrolysis is currently lacking. Consequently, their involvement should be regarded as hypothetical rather than experimentally established. In contrast, catechol-*O*-methyltransferase (COMT) has been implicated in the methylation of AVN-C, generating the structurally related AVN-B or methylated phenolic metabolites during intestinal metabolism (C. Chen et al., 2020; Pretorius & Dubery, 2023). These results suggest that intestinal metabolism may substantially alter the chemical forms reaching the circulation, with biological activity potentially being mediated by metabolites rather than the parent AVNs. However, the relative contribution of individual metabolic pathways to human AVN disposition remains insufficiently resolved.

Chen et al. (2020) observed that pretreatment of Caco-2 cells with the monoamine oxidase (MAO) inhibitor pargyline significantly increased AVN permeability while reducing the formation of hydrolysed metabolites, leading the authors to propose MAO involvement in AVN metabolism. Although MAO involvement has been proposed, current biochemical knowledge indicates that MAO does not catalyse amide bond cleavage. Consequently, the observed effects following pargyline treatment may reflect indirect alterations in cellular physiology rather than direct enzymatic metabolism of AVNs. However, definitive mechanistic studies are currently unavailable, and the underlying mechanism therefore remains unresolved. This example highlights the need for careful mechanistic validation, as apparent transport effects may reflect indirect cellular responses rather than direct enzymatic metabolism and reminds us that *in vitro* transport studies can overestimate systemic bioavailability.

## 5 Small intestinal phase II – functional modulation

Once present in the intestinal lumen, AVNs may interact with dietary macromolecules and digestive enzymes in ways that could influence starch digestion and subsequent glucose availability (C. Chen et al., 2024; Mathews & Chu, 2025). Because these interactions are strictly conditional upon thermal processing, starch source, and specific molecular structures, undergoing gelatinisation (such as heating to 95 °C) is required to alter the starch crystal matrix and unlock binding sites, allowing residual AVN-starch complexes to form. These complexes limit pancreatic α-amylase access to starch chains through hydrophobic interactions and hydrogen bonding, which vary based on the structural type of the starch (*e.g.*, amylose *versus* amylopectin) and the hydrophobicity of the specific AVN isoform (such as the highly binding AVN-A and AVN-B *versus* the less hydrophobic AVN-C). This conditional mechanism slows enzymatic hydrolysis and increases resistant starch (RS) content by up to 4.3-fold for specific isoforms. Furthermore, AVNs may also bind to amylase molecules in a noncatalytic, putative manner, further reducing starch hydrolysis (Aleixandre & Rosell, 2022). This highlights that AVNs are not merely passive antioxidants but function as context-dependent matrix modulators, a property often overlooked when bioactivity is assessed solely based on systemic concentrations.

Beyond starch modulation, AVNs inhibit the transport of free glucose across the intestinal epithelium (Mathews & Chu, 2025). Cell-based and heterologous expression studies indicate that AVNs can inhibit two primary intestinal glucose transporters: sodium-glucose transport protein 1 (SGLT1) and glucose transporter 2 (GLUT2). Heterologous expression studies show AVNs can fully block GLUT2-mediated glucose uptake and reduce SGLT1-mediated uptake by 78–84%, in a dose-dependent manner (Zhouyao et al., 2022). Major isoforms such as AVN-B and AVN-C exhibit half-maximal inhibitory concentration values of 161.8 μM and 227.8 μM, respectively. To evaluate whether these concentrations are theoretically achievable after oat consumption, Zhouyao et al. (2022) estimated intestinal AVN exposure based on several physiological assumptions. This calculation assumes consumption of approximately 100 g of oatmeal containing 13 mg AVNs, which corresponds to approximately 41–43 μmol of AVNs based on molecular weights of major isoforms (∼300–330 g/mol) (Zhang, Ni, et al., 2020; Zhouyao et al., 2022). Assuming dilution into approximately 300 mL of intestinal fluid, including ingested water and gastrointestinal secretions, the resulting theoretical luminal concentration is approximately 150 μM. This estimation is consistent with physiological models describing intestinal fluid availability during digestion. However, AVN content in oats is highly variable, depending on genotype, cultivation conditions, and processing, with reported concentrations ranging from approximately 2.1 to 407.4 μg/g (Alemayehu et al., 2023; Woolman & Liu, 2022). Therefore, this value should be interpreted as an estimated exposure scenario rather than a universal physiological concentration. Furthermore, this calculation assumes efficient release of AVNs from the food matrix and does not represent the concentration of intact compounds available for absorption. As discussed previously, AVN accessibility is influenced by starch–AVN interactions, protein associations, and chemical instability under intestinal alkaline conditions. Nevertheless, transporter inhibition occurs at the luminal-facing surface of intestinal epithelial cells and therefore may occur during transient exposure to locally elevated AVN concentrations before extensive degradation, metabolism, or absorption. This distinction is particularly important because AVNs exhibit low systemic bioavailability, yet may still exert biological effects through local gastrointestinal mechanisms. Based on these assumptions, a transient luminal concentration approaching 150 μM could theoretically achieve biologically relevant inhibition of glucose transporters, corresponding to approximately ≥75% inhibition of SGLT1 and complete inhibition of GLUT2 in experimental models (Zhouyao et al., 2022). However, direct confirmation of this mechanism requires human intervention studies measuring free AVN concentrations within the intestinal lumen during digestion. Thus, Phase II highlights that AVNs may contribute to postprandial glucose regulation through local intestinal mechanisms rather than systemic antioxidant activity alone. Their functional effects are likely determined by the combined influence of matrix interactions, isoform composition, processing conditions, and transient luminal availability, factors that should be considered when designing oat-based functional food interventions. Overall, Phase II suggests that AVNs may exert important local digestive effects, with their functional relevance likely determined by matrix interactions, isoform composition, and transient luminal concentrations rather than systemic exposure alone.

## 6 Absorption

**Fig. 2:**
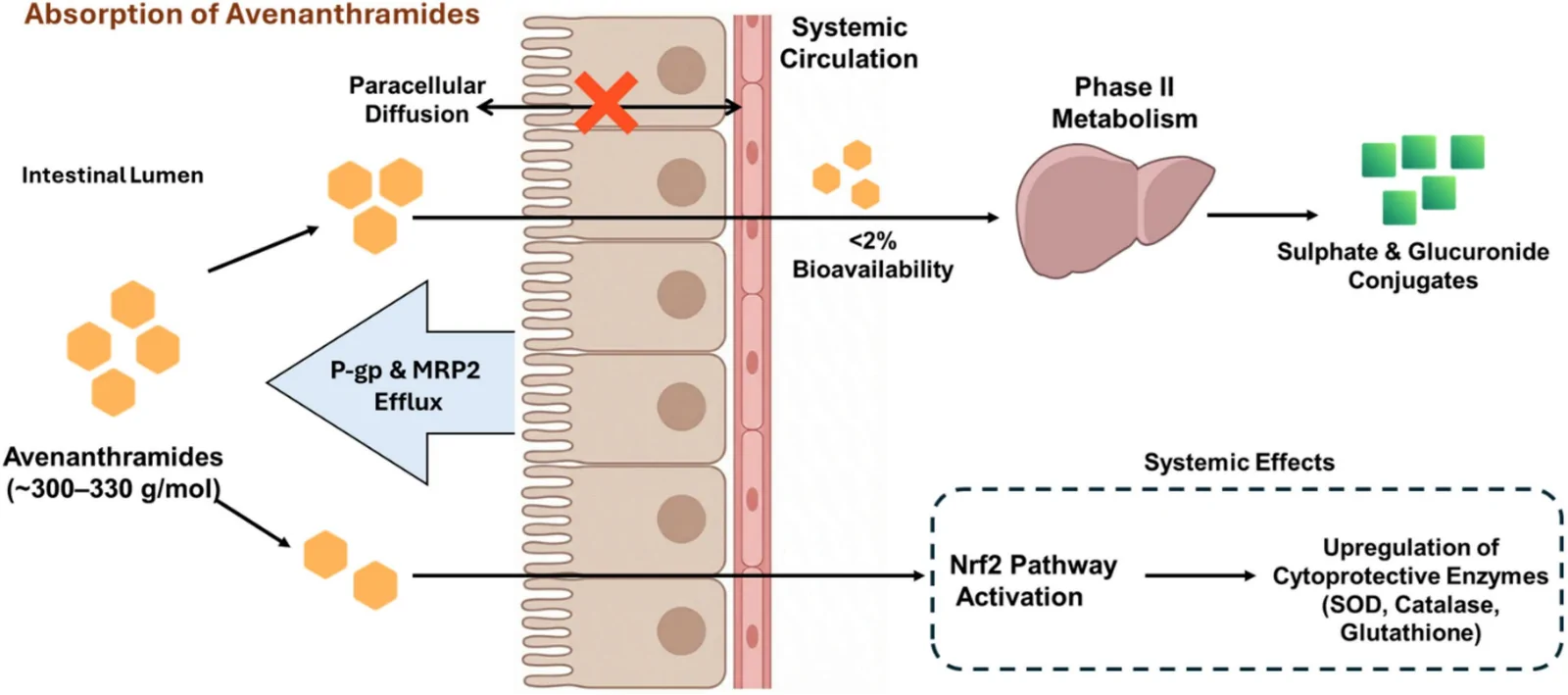
Barriers to intestinal absorption and mechanisms of systemic bioactivity for oat avenanthramides (AVNs). Absorption is restricted by molecular weight (∼300–330 g/mol), limited paracellular diffusion, and active P-gp/MRP2-mediated efflux, resulting in <2% bioavailability. Absorbed AVNs undergo rapid Phase II metabolism into sulphate and glucuronide conjugates. Despite low systemic concentrations, bioactivity is achieved through nuclear factor erythroid 2-related factor 2 (Nrf2) pathway activation, which upregulates cytoprotective enzymes (SOD, catalase, and glutathione) to restore redox homeostasis.

Intestinal absorption of native AVNs is inherently limited by their physicochemical and structural characteristics. Their relatively high molecular weight (∼300–330 g/mol) and covalent amide bond restrict carrier-mediated transport across the small intestinal epithelium (Zarrelli & Longobardo, 2025) (Fig. 2). Unlike simpler phenolic acids (*p*-coumaric, caffeic, ferulic; MW 160–200 g/mol), which are efficiently transported *via* monocarboxylate transporters, AVNs primarily rely on paracellular diffusion, a slow pathway sensitive to molecular size and polarity (Armeni et al., 2025; C. Chen et al., 2020; Hakkola et al., 2021; Maliar et al., 2023). Absorption is further constrained by active efflux mechanisms, as epithelial transporters such as P-glycoprotein (P-gp) and multidrug resistance-associated protein 2 (MRP2) export AVNs from enterocytes back into the intestinal lumen (C. Chen et al., 2020). Low aqueous solubility and partial enzymatic modification further reduce passive diffusion. These results indicate that the low systemic bioavailability of intact AVNs may not fully capture their overall physiological potential, particularly because substantial interactions occur within the gastrointestinal lumen prior to absorption. Indeed, human pharmacokinetic studies consistently report plasma recovery of intact AVNs below approximately 2% of the ingested dose, whereas substantially greater quantities remain within the gastrointestinal tract during digestion (Rana et al., 2025; Shlisky et al., 2025). This quantitative disparity supports the concept that local gastrointestinal exposure is considerably greater than systemic exposure following dietary intake, with most ingested AVNs remaining within the gastrointestinal tract, where they continue to interact locally with digestive enzymes or the microbiota (Campbell, 2021; Yu et al., 2022). Nevertheless, only a limited number of controlled human intervention studies have simultaneously quantified luminal AVN concentrations, circulating metabolites, and physiological outcomes, making it difficult to define the relative contributions of local and systemic mechanisms to AVN bioactivity. The small fraction that enters systemic circulation undergoes rapid Phase II metabolism in enterocytes and the liver, forming predominantly sulphate and glucuronide conjugates that dominate circulating species (Hakkola et al., 2021; Roumani et al., 2020). These conjugated forms may retain biological activity or function as reversible reservoirs of active metabolites. From a functional perspective, this reinforces the concept that AVNs exert their primary effects locally in the gut, with systemic circulation serving more as a secondary or modulatory pathway rather than the main site of action.

Even at very low systemic concentrations, AVNs can trigger indirect antioxidant mechanisms, particularly *via* nuclear factor erythroid 2-related factor 2 (Nrf2) activation and upregulation of cytoprotective enzymes, including superoxide dismutase, catalase, and glutathione (Baik et al., 2025; Paudel et al., 2021; Zhang, Ni, et al., 2020). These responses restore redox homeostasis and protect tissues from oxidative damage, demonstrating that systemic bioactivity can occur independently of direct radical scavenging (Favari et al., 2024; Hafez-Ghoran et al., 2025; Kim, Kim, et al., 2021). Evidence from animal models further indicates limited distribution of absorbed AVN species and metabolites to peripheral tissues such as liver, heart, and skeletal muscle, although quantitative human data remain scarce (Baik et al., 2025; Sen et al., 2025; Yu et al., 2022). Critically, this reinforces the idea that AVNs' functional relevance is dominated by local intestinal interactions and metabolic modulation rather than widespread systemic exposure.

## 7 Colonic phase

**Fig. 3:**
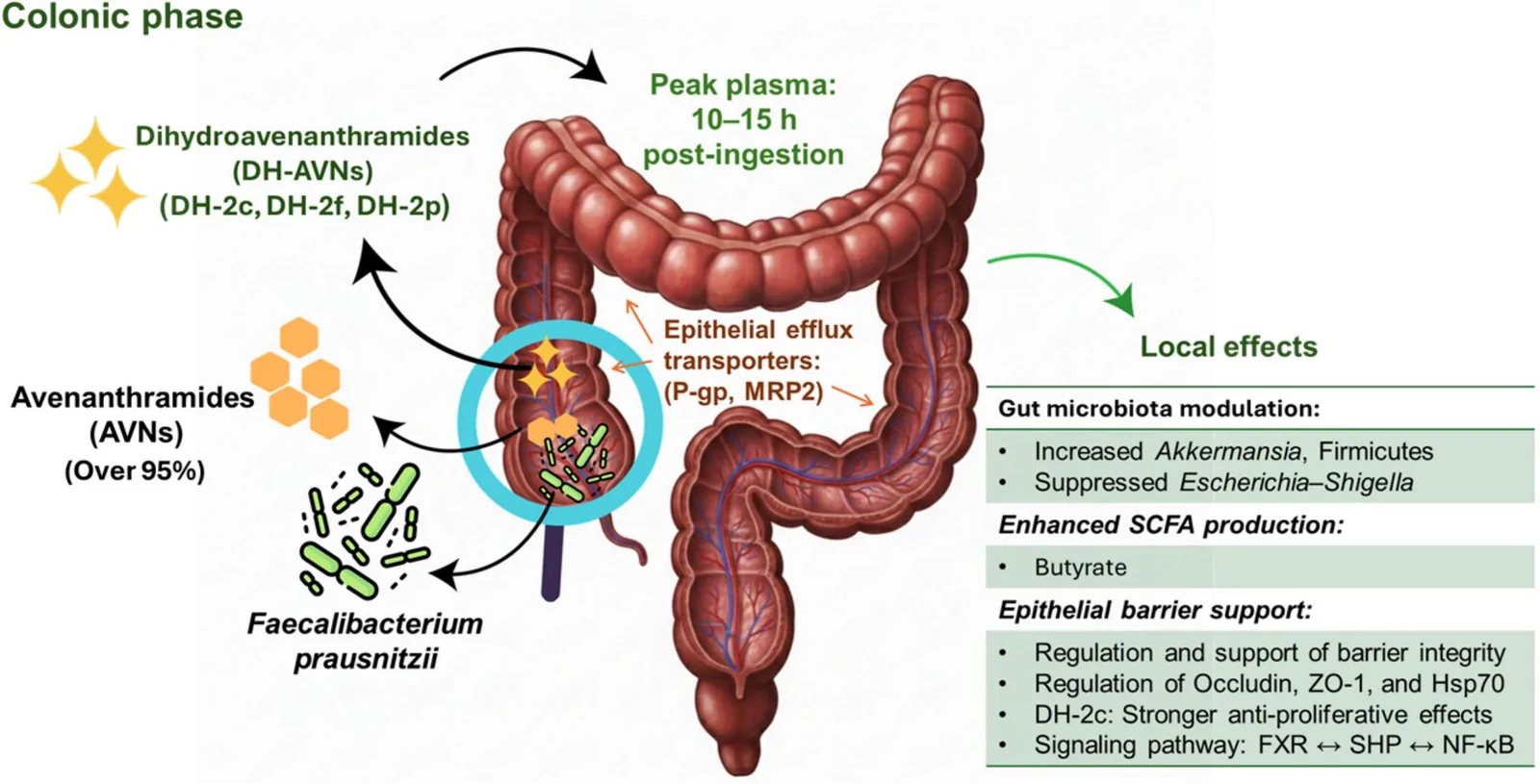
Colonic microbial biotransformation and local physiological effects of avenanthramides (AVNs). Over 95% of ingested AVNs reach the colon, where *Faecalibacterium prausnitzii* mediates their biotransformation into dihydroavenanthramides (DH-AVNs), reaching peak plasma levels 10–15 h post-ingestion. Local effects include gut microbiota modulation (increased *Akkermansia*; suppressed *Escherichia*–*Shigella*), enhanced butyrate production, and intestinal barrier support *via* regulation of Occludin, ZO-1, and Hsp70 through the FXR ↔ SHP ↔ NF-κB signalling pathway.

Clinical pharmacokinetics in healthy adults show that upper gastrointestinal absorption of intact parent AVNs is exceptionally low, with total plasma content at the maximum plasma concentration representing less than 1% of the oral dose (0.06% to 0.65% across variants) after a transit time of 1.5 to 2.3 h to reach peak blood levels (C.-Y. O. Chen et al., 2007). This aligns with wider polyphenol literature, indicating that approximately 95% of dietary phenolic compounds escape upper gastrointestinal absorption (Rana et al., 2025). Consequently, it is estimated that the vast majority of ingested AVNs reach the colon largely undigested, where the gut microbiota serves as the primary agent of biotransformation (Fig. 3). Specifically, microbial reduction of the C7′–C8′ double bond generates DH-AVNs, including DH-2c, DH-2f, and DH-2p, with *Faecalibacterium prausnitzii* identified as the critical species mediating this conversion (Campbell, 2021; *P.* Wang et al., 2021). This underscores that the colonic microbiota is not a passive site of metabolism but an active determinant of AVN functionality, effectively converting low-bioavailability parent compounds into highly bioactive metabolites.

Inter-individual variability in DH-AVN production arises from differences in microbial composition, host metabolic enzymes, and physiological factors. Direct functional studies using pure cultures and gnotobiotic mouse models have demonstrated that *Faecalibacterium prausnitzii* is capable of and sufficient for the biotransformation of AVNs into DH-AVNs, confirming a causal role under controlled experimental conditions (Campbell, 2021). However, whether *F. prausnitzii* represents the sole or dominant determinant of DH-AVN production in humans remains uncertain, as the specific microbial reductase enzyme(s) responsible for this conversion have not yet been identified, and other microbial factors may contribute to this pathway (Armeni et al., 2025; *P.* Wang et al., 2021). Clinical studies distinguish metabolizers, who efficiently produce DH-AVNs (over 50% of individuals), from non-metabolizers, who generally exhibit reduced capacity for DH-AVN production and have been associated with lower *F. prausnitzii* abundance (∼20%) (*P.* Wang et al., 2021); however, this relationship remains preliminary and is not consistently predictive of systemic exposure across human cohorts. Host enzymes and epithelial efflux transporters, including P-gp and MRP2, further modulate systemic exposure by influencing DH-AVN availability during colonic transit (C. Chen et al., 2020). Age, diet, and overall microbiome composition also shape both the magnitude and kinetics of microbial conversion (Favari et al., 2024; Roumani et al., 2020). This highlights the functional relevance of “metabotypes,” as AVN efficacy is highly personalised, underscoring that population-level recommendations may overlook critical inter-individual differences in colonic processing.

Notably, interventions such as prebiotic supplementation (*e.g.*, inulin) or exercise have been reported to increase *F. prausnitzii* abundance in individuals undergoing exercise training, patients with type 2 diabetes, and populations with lower anxiety or depression scores. However, whether these interventions can enhance AVN responsiveness or convert individuals from a non-metabolizer to a metabolizer phenotype remains unknown and should be regarded as an important direction for future research rather than an established therapeutic strategy. Evidence from *in vitro* and animal studies suggests that microbial-derived DH-AVNs may exhibit greater biological activity than their parent compounds. DH-2c, in particular, has demonstrated significantly stronger growth-inhibitory and pro-apoptotic effects against HCT-116 human colon cancer cells than its parent compound, AVN-2c, at equivalent concentrations (*P.* Wang et al., 2015). Unlike parent AVNs, which are absorbed earlier in the gastrointestinal tract, DH-AVNs are produced and absorbed in the colon. Consequently, DH-AVNs reach peak plasma concentrations approximately 10–15 h after ingestion, compared with approximately 4 h for intact AVNs. These results support the concept that the colon functions as a metabolic activation site, where parent AVNs are converted into physiologically relevant metabolites with systemic potential. Nevertheless, the physiological relevance of these results remains to be fully established. Current evidence supporting enhanced DH-AVN bioactivity is derived predominantly from cell-based and animal models, which may not fully recapitulate the complexity of the human gastrointestinal environment. Furthermore, inter-individual differences in microbial metabotypes mean that approximately 20% of individuals exhibit limited capacity to produce DH-AVNs, potentially restricting systemic exposure to these metabolites (*P.* Wang et al., 2021). Consequently, it remains unclear whether the nanomolar concentrations of DH-AVNs detected in human circulation are sufficient to reproduce the biological effects observed under the substantially higher concentrations typically employed in experimental models.

Beyond direct bioactivity, preclinical studies suggest that AVNs and DH-AVNs are associated with alterations in the colonic microbiota, including increased relative abundance of beneficial taxa such as *Akkermansia*, Firmicutes, *Roseburia*, *Blautia*, and *Lachnospiraceae*, together with reduced abundance of potential pathogens such as Escherichia–Shigella (Rana et al., 2025; Ye et al., 2025). This microbial reshaping has been associated with enhanced short-chain fatty acid (SCFA) production, particularly butyrate, which fuels colonocytes and supports intestinal barrier integrity (Liu et al., 2022). Consistent with these microbiota-associated changes, AVNs have been reported to enhance intestinal barrier function in several preclinical models. In ovalbumin-induced food allergy models, AVN treatment increased the expression of the tight-junction proteins Occludin, Zonula Occludens-1 (ZO-1), and Claudin-1, whereas in high-fat diet (HFD)-induced obesity models, AVN-B increased Occludin and MUC2 expression in the terminal ileum (P. Liu et al., 2022; Y. Liu et al., 2024). In dextran sulfate sodium-induced colitis models, AVN-C alleviated colonic injury by modulating bile acid metabolism through the Farnesoid X Receptor–Small Heterodimer Partner–Nuclear Factor kappa B (FXR-SHP-NF-κB) signalling pathway, thereby improving barrier integrity and reducing inflammatory mediators (Ye et al., 2025). These local intestinal effects have been associated with broader cardiometabolic improvements in HFD-induced obesity models, including reduced weight gain, hepatic steatosis, and dyslipidaemia, while anti-atherosclerotic activity has been demonstrated separately through inhibition of matrix metalloproteinase-9 (MMP-9)-mediated vascular smooth muscle cell migration and reduced atheroma formation in low-density lipoprotein receptor-deficient mice (Park et al., 2021; Thomas et al., 2018). However, these mechanistic observations originate predominantly from disease-specific rodent and cell-based models. Therefore, the relative contribution of individual pathways to the health effects of dietary AVNs in humans remains uncertain, and well-controlled human intervention studies are required to establish their physiological relevance. Most evidence is derived from 16S rRNA sequencing and correlation-based analyses in animal models, and the contribution of individual taxa to AVN-mediated health effects remains to be confirmed using functional approaches, such as faecal microbiota transplantation, together with well-controlled human intervention studies. Critically, this suggests that the biological impact of AVNs may be mediated, at least in part, through local colonic and microbial interactions rather than direct systemic absorption, reinforcing a paradigm shift from *in vitro*-centric antioxidant assumptions to digestion- and microbiota-driven functionality.

## 8 Evidence supporting the proposed digestion-stage mechanisms

The digestion-stage framework presented in this review integrates evidence derived from food chemistry, simulated gastrointestinal digestion, cell culture experiments, animal studies, and the limited number of human intervention and pharmacokinetic studies. To facilitate interpretation of the proposed framework, Table 3 summarises the principal digestion-stage mechanisms proposed in this review, together with the highest level of supporting evidence and the main limitations of the currently available literature. As shown, the strength of evidence varies substantially across mechanisms. While low systemic bioavailability and microbial biotransformation are supported by human pharmacokinetic studies, many functional mechanisms remain based predominantly on *in vitro* and preclinical evidence and therefore require confirmation in well-designed human intervention trials.

**Table 3:** Summary of the current evidence supporting the proposed digestion-stage mechanisms of avenanthramides (AVNs).

Proposed mechanism	Highest level of supporting evidence	Main evidence available	Main limitation
Matrix association	*In vitro* digestion + food chemistry	Analytical chemistry, food matrix studies, simulated gastrointestinal digestion	Limited *in vivo* validation of AVN–matrix interactions during human digestion
Gastric stability	*In vitro* digestion	Simulated gastrointestinal digestion and analytical chemistry	Few human studies confirming stability under physiological conditions
Phase I metabolism	Cell studies	Caco-2 * transport studies and analytical chemistry	Responsible metabolic enzymes remain incompletely identified and physiological relevance is uncertain
Starch digestion and resistant starch modulation	*In vitro* digestion	Simulated digestion studies examining AVN–starch interactions	Effects depend on starch source, processing conditions and experimental model; human confirmation lacking
Glucose transporter inhibition (SGLT1/GLUT2)	Cell models	Caco-2 cells and heterologous transporter-expression systems	No direct measurements of intestinal AVN concentrations or transporter inhibition in humans
Low systemic bioavailability	Human intervention studies	Human pharmacokinetic studies supported by cell transport experiments	Limited number of controlled intervention studies simultaneously measuring pharmacokinetics and physiological outcomes
DH-AVN production	Human pharmacokinetics + microbial culture studies	Human studies, microbial culture experiments and animal models	Microbial enzymes responsible for DH-AVN formation remain unidentified, and interindividual variability is incompletely understood
Gut microbiota modulation	Animal studies	Animal models and *in vitro* fermentation studies	Human evidence is limited, and most studies establish associations rather than causality

* Note: Caco-2 = Human epithelial colorectal adenocarcinoma cell line; DH-AVN = Dihydro-avenanthramide; SGLT1, sodium-glucose cotransporter 1; GLUT2, glucose transporter 2.

## 9 Gaps and future directions

Despite growing insights into AVN digestion and metabolism, translating this knowledge into functional food design remains challenging due to gaps in methodology, modelling, and industrial application. Current understanding of AVN–matrix interactions relies mainly on FTIR spectroscopy, supported by limited NMR analyses and molecular docking. While these approaches provide valuable structural insights, FTIR offers only indirect evidence of binding, NMR is constrained by the limited availability of purified AVN standards, and molecular docking predictions require experimental validation. Analytical limitations constrain understanding, particularly the scarcity of authentic standards for minor isoforms, such as A-type avenalumic acid derivatives and glycosylated hexosides, which are often overlooked in favour of AVN-A, AVN-B, and AVN-C. Geometric E/Z isomers, despite their potentially higher activity, are rarely distinguished, and inconsistent extraction protocols hinder comparability across studies.

Biological models also fail to capture the complexity of AVN functionality. Interactions between AVNs and starch or protein significantly influence both AVN bioaccessibility and starch digestibility, yet these are rarely incorporated into *in vitro* systems. Furthermore, whether these interactions are reversible or irreversible during gastrointestinal digestion remains unclear. Although strong AVN binding to gelatinised starch has been reported, the stability of these complexes under physiological conditions and their effects on bioaccessibility are still poorly understood. Microbial biotransformation in the colon, including the production of DH-AVNs by *F. prausnitzii* and metabotype-dependent variability, is insufficiently represented, limiting predictions of individualised outcomes. Synergistic effects with other oat components, such as β-glucans and proteins, remain underexplored, leaving the full potential of whole-food functionality unclear.

Translationally, the impact of industrial processing on AVN stability and AVN-starch complexes is poorly understood, and practical methods to recover bound AVNs from oat side-streams are underdeveloped. Low oral bioavailability (<2%) highlights the need for advanced delivery systems, including encapsulation or nanoparticles, to ensure consistent functional dosing. Moreover, most evidence supporting AVN–matrix interactions is derived from *in vitro* digestion models and animal studies, whereas well-controlled human intervention studies confirming their formation and physiological relevance remain scarce. Breeding programs also have an opportunity to target genotypes with higher AVN content or greater responsiveness to natural elicitors like germination.

Addressing these gaps requires integrated strategies that combine standardised analytical methods, biologically relevant models accounting for matrix interactions and microbial metabolism, and scalable industrial approaches. Such efforts will enable the rational design of oat-based functional foods with predictable health benefits.

## 10 Conclusion

Current evidence suggests that AVNs may be conceptualised as promising multi-stage digestive modulators rather than merely bioactive compounds. Their primary physiological impact may arise from localised digestive and microbial mechanisms rather than systemic circulation of intact compounds. Their functionality appears to depend on release from the oat matrix, chemical stability during gastrointestinal transit, interactions with macronutrients, and metabotype-dependent microbial biotransformation in the colon. These processes are proposed to contribute to localised digestive effects, glucose metabolism, and systemic health outcomes, highlighting a complex interplay between food structure, chemical fate, and host–microbe interactions. Current literature is limited by reductionist approaches, insufficient representation of matrix effects, and under-characterisation of minor isoforms and geometric isomers. This review proposes that these gaps can be addressed through standardised analytical techniques, biologically relevant digestion models, and scalable industrial strategies, thereby enabling the development of AVN-targeted functional foods. However, many components of this proposed framework remain hypothetical and require confirmation through well-designed human intervention studies incorporating physiologically relevant digestion models, microbiome characterisation, and clinically meaningful functional outcomes. Such studies will be essential to establish the physiological relevance of AVN digestion-stage mechanisms and to determine their potential contribution to evidence-based dietary interventions for cardiometabolic health, intestinal barrier function, and microbiota-mediated metabolic regulation.

## CRediT authorship contribution statement

**Nima Mohammadi:** Writing – review & editing, Writing – original draft, Visualization, Validation, Software, Methodology, Investigation, Formal analysis, Data curation, Conceptualization. **Amalia G.M. Scannell:** Writing – review & editing, Writing – original draft, Validation, Supervision, Funding acquisition. **Marilú Andrea Silva-Espinoza:** Writing – review & editing, Writing – original draft, Validation, Supervision, Resources, Project administration, Funding acquisition.

## Funding

This research was supported by the Department of Agriculture, Food and the Marine (DAFM), Ireland, through the HOPE Project (Grant No. 2023RP1031). No additional external funding was received.

## Declaration of competing interest

The authors declare the following financial interests/personal relationships which may be considered as potential competing interests: Marilu Andrea Silva Espinoza reports financial support was provided by Government of Ireland Department of Agriculture Food and the Marine. If there are other authors, they declare that they have no known competing financial interests or personal relationships that could have appeared to influence the work reported in this paper.

## Data Availability

No data was used for the research described in the article.
